# Immunogenicity correlation in cynomolgus monkeys between Luminex‐based total IgG immunoassay and pseudovirion‐based neutralization assay for a 14‐valent recombinant human papillomavirus vaccine

**DOI:** 10.1002/jmv.27763

**Published:** 2022-04-21

**Authors:** Lei Bei, Xiao Zhang, Dan Meng, Shuman Gao, Jilei Jia, Dandan Zhao, Chunxia Luo, Xuefeng Li, Hongbin Qiu, Liangzhi Xie

**Affiliations:** ^1^ School of Basic Medicine Jiamusi University Jiamusi Heilongjiang China; ^2^ Beijing Engineering Research Center of Protein and Antibody Sinocelltech Ltd. Beijing China

**Keywords:** correlation, HPV, immunogenicity, Luminex‐based total IgG assay (LTI), pseudovirion‐based neutralization assay (PBNA), vaccine, VLP

## Abstract

A new virus‐like particle based vaccine covering 14 types of high‐risk and disease‐inducing human papillomavirus (HPV) can offer better coverage against HPV‐induced diseases, particularly cervical cancers. However, the assessment of immunogenicity of the vaccine is an important task, representing not only its significant clinical characteristics, but also a major challenge, in terms of both the suitability of methods and the clinical sample testing throughput supporting clinical development. This work covers the development and evaluation of a method based on Luminex technology (a coded‐bead and flow‐cytometric approach) to assess the HPV‐type specific total immunoglobulin G (IgG). This method can evaluate the antibodies in sera post immunization against multiple types of HPV simultaneously (i.e., with multiplexing capability), save time and cost, and improve test throughput with higher sensitivity and precision than the classical, plate‐based enzyme‐linked immunoassay and competitive Luminex‐based immunoassays. Using cynomolgus monkeys as model, we demonstrated the good correlation between the results from the pseudovirion‐based neutralization assay (PBNA), and the Luminex‐based total IgG assay, supporting that the latter method can be considered as a viable, dependable replacement method for the PBNA supporting immunogenicity evaluation of HPV vaccine in preclinical development and clinical investigation.

## 1 INTRODUCTION

Human papillomaviruses (HPVs) are double‐stranded DNA viruses that can infect human epithelial cells, leading to many kinds of diseases, including cervical cancers and external genital warts. It is well known that persistent infection of HPVs can first lead to cervical intraepithelial neoplasias with progressive grades and then to cervical cancer. In fact, 99.7% patients with cervical cancer could be detected with evidence of HPV infection.^1^ The cause and consequence of HPV infection and cervical cancer are so clear that the researcher who originally discovered the connection has received the Nobel Prize in medicine.^2^

The majority of HPV types associated with human cervical cancers are from HPV16, 18, 31, 33, 35, 39, 45, 51, 52, 56, 58, 59, and likely 68.^3^ International Agency for Research on Cancer had classified HPV into the low‐risk and high‐risk types, and the high‐risk types were further classified into 3 classes, the first class of which (carcinogenic) includes 12 types, i.e., HPV16, 18, 31, 33, 35, 39, 45, 51, 52, 56, 58, and 59.^4^

The discovery of HPV causing cervical cancers paved the road for vaccine development. Over the past decade or so (since 2006), virus‐like particle (VLP) based HPV vaccines had become the major product to prevent HPV infection and related diseases. Those VLPs are made by recombinant HPV L1 capsid proteins. The capsid structures of VLPs in the vaccines are highly similar to those of the natural virus particles so that they not only have the good immunogenicity to induce high titer of neutralization antibodies against individual HPV types but also offer long‐term protection against HPV‐induced diseases. With the absence of a viral genome, recombinant VLP‐based vaccines not only have proven efficacy but also have a good safety profile. Until June of 2021, there are 4 types of HPV vaccines which are all L1‐protein based VLP vaccines, approved for marketing, Gardasil® (approved in 2006),^5^ Cervarix® (approved in 2007),^6^ Gardasil 9® (approved in 2014)^7^ and Cecolin® (approved in China in 2019).^8^ Among these vaccines, Gardasil 9® has the most extensive protection coverage which is about 89.7%,^9^ covering both carcinogenic types (HPV16, 18, 31, 33, 45, 52, and 58) and genital warts related types (HPV6 and 11).

There are still many HPV vaccine candidates in various stages of the clinical development, including the one developed by Sinocelltech, Ltd. with 14 HPV types (i. e., HPV6, 11, 16, 18, 31, 33, 35, 39, 45, 51, 52, 56, 58, and 59). This VLP‐based HPV vaccine has the potential to offer the best coverage against HPV‐induced diseases to date, with the potential cervical cancer protection rate worldwide as high as 95.4%.^9^ This new vaccine covers both the genital warts‐related types (HPV6 and 11) and high‐risk carcinogenic 12 types classified by World Health Organization (WHO). It adopted the expression system of transfected baculovirus‐infected insect cells, which is a similar approach as those for marketed vaccines, Cervarix® and Flublock®. This 14‐valent HPV vaccine has completed all the preclinical investigations, its IND for the early clinical trial has been approved by CDE (Center for Drug Evaluation), and Phases I and II clinical investigations have started in July 2021 and October 2021, respectively.

Since there is no adequate, relevant animal model for HPV infection due to viral species‐specificity, it is recommended by WHO that the pharmacodynamics properties of an L1‐VLP‐based vaccine should be assessed through immunogenicity studies in addition to other methods defined by an individual national regulatory authority.^10^ To evaluate the immunogenicity of HPV vaccines, there are several major methods, such as pseudovirion‐based neutralization assay (PBNA), competitive immunoassays using a set of neutralizing monoclonal antibodies (with one for each HPV type) as competitors, and direct binding IgG assays using intact and structurally sound VLPs as antigens, all of which can be used to measure antibody levels to the VLPs in postvaccination serum. These assays need to be sensitive, reproducible, simple to perform, and amenable to high‐throughput testing due to the large burden of HPV vaccine efficacy trials. Neutralization assays (specifically, the PBNA type) are considered “the gold standard” for assessing the protective potential of antibodies induced by the HPV vaccines, which are indicated in the WHO guidelines.^10^ Direct‐binding ELISA has been used for the clinical evaluation and regulatory registration of Cervarix®, which was shown to have a high correlation with PBNA with correlation coefficients ranging from 0.70 to 0.94 for HPV16 and HPV18, which has been used to support BLA and marketing approval.^11^ At the same time, the single neutralizing epitope‐based antibody inhibition ELISA was compared with the PBNA, with the correlation coefficients ranging from 0.57 to 0.96 for HPV16 and HPV18. Therefore, the GSK's direct‐binding ELISA has been established and accepted by regulators (including US Food and Drug Administration [FDA]^12^ and EMA^13^) as an excellent surrogate for measuring neutralization antibodies against HPV16 and HPV18.

In this study, for the purpose of supporting the 14‐valent vaccine clinical studies, we developed a method based on the Luminex technology to assess the HPV‐type specific total IgG with the same mechanism as that of direct‐binding ELISA. Importantly, the Luminex‐based platform (i.e., Luminex Total IgG or LTI assay) can support the evaluation of the antibodies against multiple types of HPV‐VLP simultaneously, which has the advantages of saving time and improving test throughput, reliability, and data analysis. We have demonstrated with the nonhuman primate studies that the immunogenicity data obtained from the LTI high‐throughput method correlate well with those from the PBNA method, and the LTI method can be considered a useful and efficient supplement or replacement method for supporting both the preclinical and clinical immunogenicity assessment in the development of HPV vaccines with high valency.

## 2 MATERIALS AND METHODS

### 2.1 HPV VLPs

Recombinant HPV VLP vaccine used in the preclinical study contained VLPs of HPV types 6, 11, 16, 18, 31, 33, 35, 39, 45, 51, 52, 56, 58, and 59 with 9 types overlapping with those in Gardasil 9® and the new 5 types (HPV 35, 39, 51, 56, and 59).

The 14‐valent recombinant vaccine is prepared from the purified individual VLPs of the individual major capsid (L1) proteins of 14 HPV types. First, the recombinant baculovirus for each HPV type can be acquired from the expansion in insect cells, after the construction of the recombinant plasmid containing the L1 gene of each type of HPV and transfection of baculovirus. The L1 proteins are produced by the expression and expansion of insect cells that have been infected with the baculovirus and self‐assembled into VLPs within the cells for each type. At the end of the expression, the insect cells were harvested and homogenized with the HPV VLPs isolated from the cell debris through centrifugation. The clarified supernatant for each HPV type was separately further purified through multiple steps of chromatography and filtration as well as virus inactivation and removal processes, leading to the aqueous drug substance bulk for each HPV type.

To study and confirm the structural and quality properties of the individual drug substance to be used for the 14‐valent vaccine, dynamic light scattering (DLS) was used to assess the conformational integrity of each VLP type, which was a method to obtain the particle size information by measuring the fluctuation of the scattered light intensity of the particulate sample with time. The DLS analyses of the drug substance of the 14‐valent vaccine are carried out with the results showing that the protein particle size and its distribution of each batch of drug substance meet the quality standards (data not shown). Furthermore, the tungsten‐stained transmission electron microscope (JEOL, JEM 1200EX) was also used to assess the conformational integrity and particle morphology of each VLP type. The results showed that the VLP structure of each batch of the vaccine drug substance was fairly uniform with particle size about 60 nm in diameter. The structural features and protein particle size are consistent within the samples and among different production batches. The typical figures of electron microscope were shown in Figure S1.

To evaluate the type‐specific conformation of the VLPs used in this study, we have developed 14 type‐specific and neutralizing monoclonal antibodies to identify each type of VLPs. It is well known that type specific and neutralizing antibody targets conformational epitopes.^14^, ^15^ Each batch of 14‐valent VLPs was verified using these specific antibodies by enzyme‐linked immunosorbent assay (ELISA) before using for immunization (as shown in Figure S2).

For VLP cross‐reactivity testing, EC_50_ of immunized serum by single type VLP was evaluated using 14 types of pseudovirus separately by the PBNA method. The data showed good specificity of VLPs, as shown in Table S1. There was no obvious cross‐reaction between different types of VLPs except for a slight cross‐reaction between HPV 6 and HPV 11. The cross‐reaction of HPV 6 and HPV 11 came from the cross‐protection between HPV 6 and HPV 11. As reported by Christensen ND,^16^ HPV 6 and HPV 11 share high sequence homology (>90%) and contain certain shared neutralizing epitopes that contributed to the cross‐reaction.

### 2.2 Formulation of testing sample

The individually expressed, separated, and purified VLP drug substances are absorbed on the preformed aluminum‐phosphate‐based adjuvant. Then, combining the adjuvant‐adsorbed VLPs of each HPV type in a predefined ratio to produce the final vaccine with formulation buffer containing histidine, sodium chloride, and polysorbate 80 post fill and finish process.

### 2.3 Animal immunization

Cynomolgus monkeys were immunized via intramuscular injection with different doses and frequencies for various study objectives. In this study, we focused on the correlation between the two methods of immunogenicity assessment, namely the PBNA and LTI methods, and the data of both HPV type‐specific total IgG concentration and neutralizing antibody titer were collected, analyzed, and compared. The vaccination method and sampling time points are shown in Table 1. In Study #1, the animals were vaccinated with a low dose (37 μg total antigens), and in Study #2, the animals were vaccinated with high doses (370 and 1110 μg total antigens) which are at least 10 times the low dose. The samples used for vaccination in Study #1 were produced with higher quality by an optimized manufacturing process, compared with the samples used in Study #2.

**Table 1:** The vaccination and sampling information for PBNA and LTI evaluation

Study	Number of animals	Dose (μg)	Dosing frequency	Serum collection timepoint (day)
#1	6	37	0, 1, 3 M	15, 31, 45, 59, 87, 91, 105, 119
	6	37	0, 1, 3 M	
#2	10	370	0, 3, 6, 9 W	42
	10	1110	0, 3, 6, 9 W	42
	4	370	0, 3, 6, 9 W	71, 92
	4	1110	0, 3, 6, 9 W	71, 92

*Note*: M = month, W = week.

Abbreviations: LTI, Luminex Total IgG; PBNA, pseudovirion‐based neutralization assay.

### 2.4 Pseudovirion‐based neutralization assay

**Figure 1:**
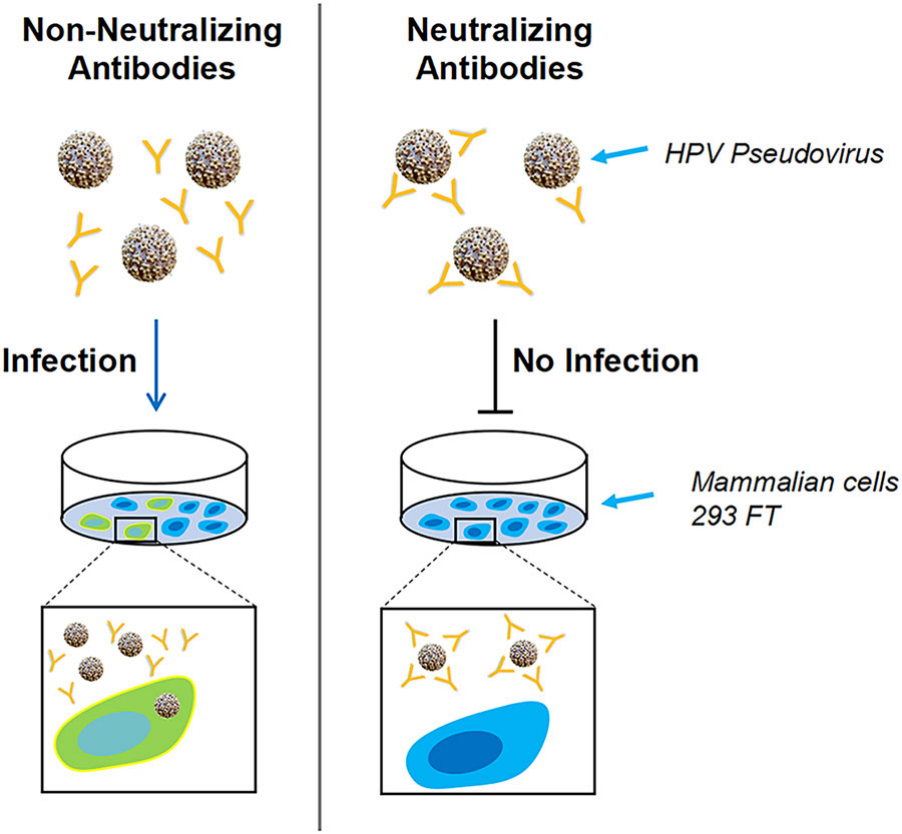
Method principle diagram of PBNA. PBNA, pseudovirion‐based neutralization assay

The HPV PBNA, which was developed independently by Sinocelltech Ltd., was utilized in this study to investigate the immunogenicity of the candidate vaccine in cynomolgus monkeys. Specifically, the cotransfection of mammalian cells with two HPV capsid genes, L1 and L2, together with the green fluorescent protein (GFP) plasmid produced high infectious titers of pseudovirions, which presents the surface conformational epitopes of the capsid proteins similar to those of the native virions. As shown in Figure 1, in the absence of neutralizing antibodies, 293FT cells would express GFP after being infected by the HPV pseudovirion, which could be easily observed and evaluated in a FluoroSpot Analyzer. In the presence of neutralizing antibodies, the green fluorescent signal is decreased quantitatively depending on the content of the antibody.

In PBNA, preimmunization and postimmunization sera were diluted serially and then incubated separately with each HPV type of pseudovirion to test the neutralizing‐antibody levels. Because it is difficult to cover all samples for all 14‐type HPV assays in one dilution range, a portion of samples were retested by a modified and more optimized dilution range. The mean of all test results for each HPV type was calculated in the final investigation.

The immunogenicity investigation of the vaccine was based on the 14‐subtype HPV PBNA. Transfection of 293FT cells by the type‐specific HPV pseudovirion could be neutralized by neutralizing antibodies stimulated by vaccine immunization of cynomolgus monkeys. When 293FT was cultured with the pseudovirion‐sera mixture, the number of GFP‐positive cells would decrease with the concentration increase of the neutralizing antibodies in monkeys' sera. As a result, within certain limits, the relationship between the amount of neutralizing antibodies and the number of GFP‐positive cells showed a negative linear correlation. Thus, GFP cell counts could reflect the level of HPV neutralizing antibodies in animal serum in PBNA.

Sera samples were inactivated at 56℃ for 30 min followed by a 10,000*g* centrifugation for 5 min. 293FT cells were plated 4–6 h in advance in a 96‐well cell culture flat‐bottom plates at 15,000 cells/well in 100 μl cell culture medium and then cultured at 37℃, 5% CO_2_. Preimmunization and postimmunization sera were serially diluted with five dilutions from 400 to 2500 and then mixed separately with each diluted HPV type 6, 11, 16, 18, 31, 33, 35, 39, 45, 51, 52, 56, 58, and 59 pseudovrius individually. After incubation at 2–8℃ for 1 h, the 100 μl pseudovrius‐sera mixture was transferred into pre‐plated 293FT cells and incubated for 62–96 h. All samples were tested in duplicate wells, and meanwhile, pseudovirion only and medium only wells were set as positive and negative controls, respectively.

### 2.5 Luminex‐based total IgG assay

**Figure 2:**
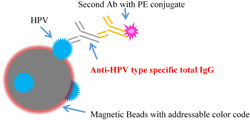
Method principle diagram of LTI. LTI, Luminex Total IgG; IgG, immunoglobulin G

The key technical core of the Luminex was to be able to address specific beads (i.e., addressable beads) by adjusting the ratio of two different fluorescent dyes to code multiple magnetic beads with different characteristics of fluorescence spectra. For the planned study, 14 kinds of commercial magnetic beads were covalently cross‐linked with an appropriate amount of specific high‐quality HPV VLPs, to obtain 14 individual beads covering 14 HPV types, i.e., one addressable bead per HPV type. The efficiency and specificity of each kind of VLP‐coupled magnetic beads were verified in the use of qualified specific anti‐HPV VLP monoclonal antibodies with high response to the target type and without cross‐interference with any other types. After quality was released, the VLP‐coupled magnetic beads were mixed and incubated with monkey serum; then, a phycoerythrin (PE)‐conjugated anti‐monkey specific secondary antibody was used as the detection reagent; finally, the complexes were detected by the Luminex 200 equipment. The fluorescence signal values (expressed as mean fluorescence intensity, MFI) were related to the amount of serum total type‐specific IgG antibody bound with the individually immobilized HPV‐VLPs on the addressable beads. The mechanism of the Luminex‐based total IgG assay is schematically shown in Figure 2.

The anti‐HPV VLP serum obtained from cynomolgus monkeys immunized repeatedly with the investigational vaccine was chosen as the standard substance to achieve the quantitative determination of total IgG in serum. This is an ideal method for the efficacy or immunogenicity testing of vaccine candidates, and for this purpose, the comprehensive methodological validation of the Luminex method for detection of total specific IgG in the serum of cynomolgus monkey has been completed. Although there were no specific acceptance criteria for the parameters in the guidelines for method validation of vaccine immunogenicity evaluation, the validation conducted referred to the guidelines and recommendations for the bioanalytical method validation of ligand‐binding assays which were generally used to support pharmacokinetic assessments with more strict criteria, and the results indicated that the method was robust and accurate, showing good appearance in all the validated parameters, such as selectivity, linearity, precision, accuracy, and stability. The LTI methods were fully validated. Briefly, the intra‐batch accuracies (%RE) of QC samples of all HPV types were −5.82%–18.81%, while their inter‐batch accuracies were 2.50%–7.99%. Precision results showed that the intra‐batch precisions (%CV) of QC samples of all HPV types were 0.16%–17.22%, and the inter‐batch precisions of the same samples were 5.31%–11.70%. The stability samples from cynomolgus monkey standard serum were prepared and proved stable with the criteria of %RE within ±20% under different conditions, including a room temperature storage for 4 h, 2–8℃ storage for 24 h, 5 freeze‐thaw cycles, and −80℃ storage for 160 days. All results of the ISR (Incurred Sample Reanalysis) test showed good reproducibility, corresponding to the %Bias within ±30%.

### 2.6 Data analysis

#### 2.6.1 PBNA

The percentage of neutralization of pseudovirion infecting 293FT cells for a given sample of a specific HPV type is calculated as follows using HPV pseudovirion positive cell counts as below.

Neutralization(%)=1–A/B

where A = Immune sera − Background_No pseudovirion_; and B = Preimmune sera − Background_No pseudovirion_.

The neutralization titers were defined as the effective concentration (EC_50_) achieving a 50% reduction of the maximal signal and were calculated with the Reed‐Muench method.

#### 2.6.2 LTI

The Luminex‐based HPV‐type specific total IgG results were shown as the concentration which was quantitated and calculated against the reference serum. The reference serum is a good alternative standard to provide a ruler for immunogenicity evaluation due to restricted international standards for all 14 HPV types. With reference to the cross‐standardization method published by Merck,^17^ the reference serum was cross‐standardized with international standards to provide a basic bridging connection with WHO international standards, which can be completed with either of the international standards (HPV 16 or HPV 18). In this study, the reference standard of monkey serum concentration for each HPV type was cross‐standardized with the reference of the WHO International Standard 05–134 HPV16 antibodies (NIBSC code: 05/134) as the standard.

The reference standard of monkey serum concentration for each HPV type was cross‐standardized with the reference of the WHO International Standard 05–134 HPV16 antibodies (NIBSC code: 05/134) as the standard. The reference serum and the Standard 05‐134 were diluted in gradient and evaluated in 3 runs of the PBNA assays. The data were calculated by the parallel lines method using CombiStats 5.0 Software to evaluate the potency or concentration of antibodies of each HPV type in the reference serum and then assigned relative potency accordingly. All potencies of 14 HPV types were expressed in mNU/ml (an arbitrary, but consistent unit), shown in Table 2, in the same way for all the LTI results of the samples.

**Table 2:** The potencies of the reference serum for 14 HPV types

Type	HPV6	HPV11	HPV16	HPV18	HPV31	HPV33	HPV45
Potency (mNU/ml)	286	477	1830	1800	470	1315	148
**Type**	**HPV52**	**HPV58**	**HPV35** a	**HPV39** a	**HPV51** a	**HPV56** a	**HPV59** a
Potency (mNU/ml)	3325	1118	934	717	4958	1620	881

a The new HPV types that are not present in Gardasil 9.

#### 2.6.3 Pearson correlation analysis

To compare the correlation of the data between PBNA and LTI methods, the Pearson correlation method was used in the analysis. In GraphPad Prism 8 software, the correlation coefficient (r) and correlation significance (two tailed)/*p* value are shown after the numeric analysis. If the significance/*p* value is less than or equal to 0.05, it proves that the correlation is significant, which means the data between PBNA and LTI are correlated. As for the correlation coefficient, the closer it is to 1, the higher extent is for the correlation between the two datasets.

## 3 RESULTS

### 3.1 Correlation analysis between PBNA and LTI in Study #1

**Figure 3:**
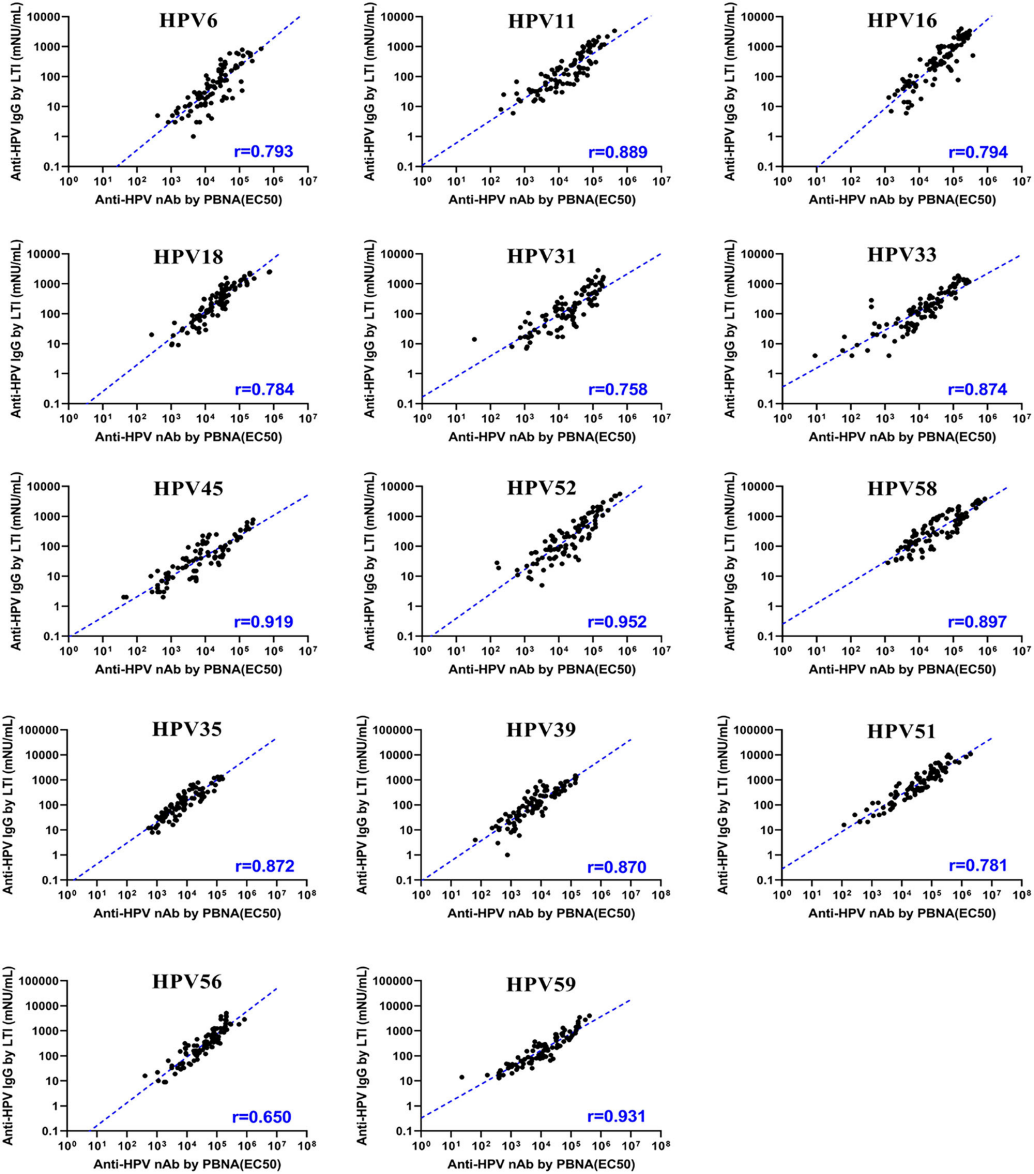
Scatter plots for the data between the PBNA titers (EC_**50**_) and specific total IgG concentrations in Study #1. The slash dotted line represents linear regression or concordance (*N* = 96). IgG, immunoglobulin G; PBNA, pseudovirion‐based neutralization assay

In Study #1, the monkeys were vaccinated with the low dose (37 μg total antigen content as shown in Table 1), with the results shown in Figure 3 (and Table S2). The results of the analysis showed that the data obtained from PBNA and LTI methods were clearly correlated for 13 HPV types (i.e., at least 0.758). Only those data from HPV 56 were shown with a relatively lower correlation coefficient, namely at 0.650 which was only slightly lower than the other types. It is worth noting that all correlations between the two methods have a clear significance with the *p* value less than 0.0001, suggesting the presence of a clear correlation, particularly considering that the datasets are still limited in size.

### 3.2 Correlation analysis between PBNA and LTI in Study #2

**Figure 4:**
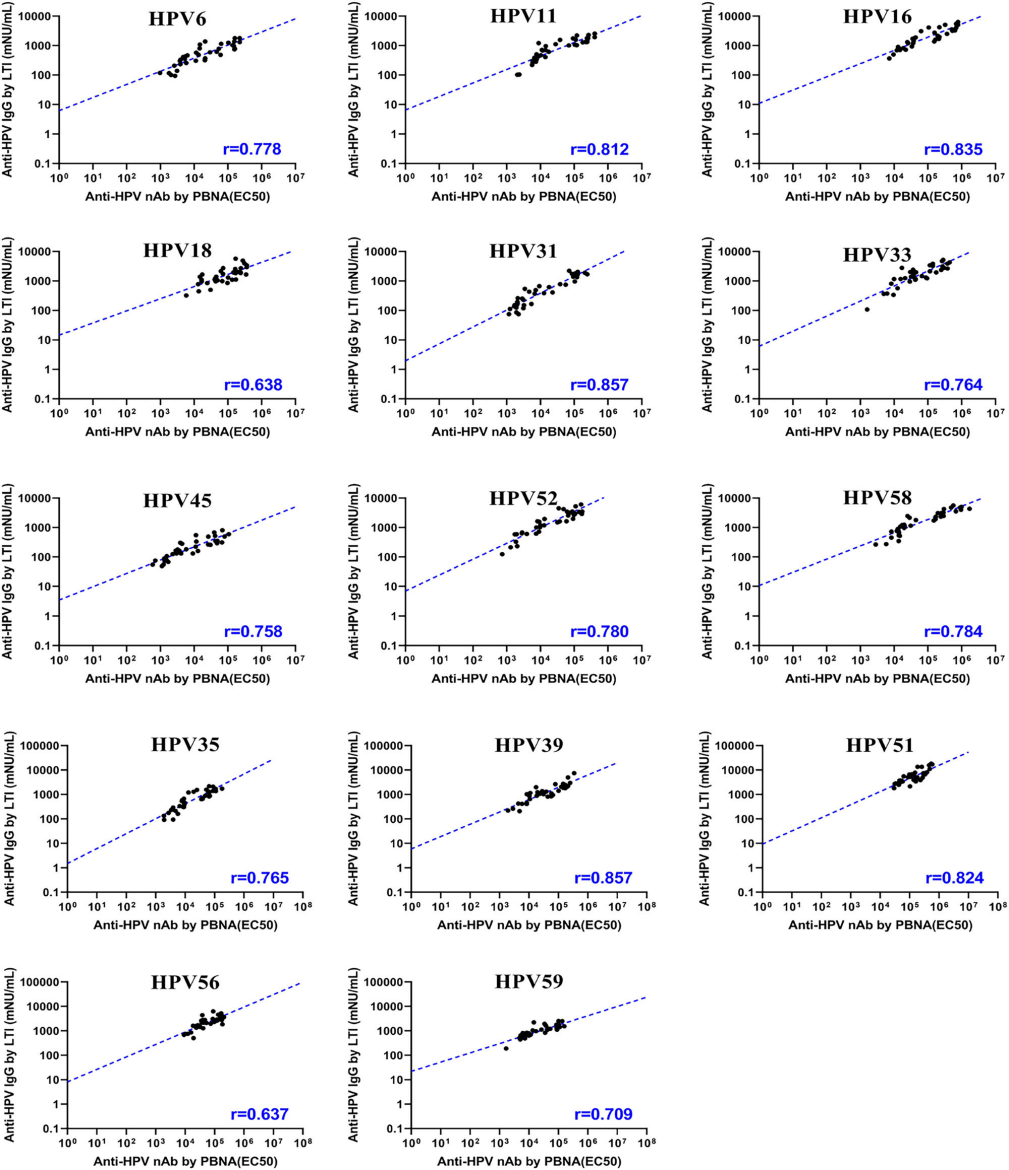
Scatter plots for the data between the PBNA titers (EC_**50**_) and specific total IgG concentrations in Study #2. The slash dotted line represents linear regression or concordance (*N* = 36). IgG, immunoglobulin G; PBNA, pseudovirion‐based neutralization assay

The correlation of the immunogenicity results between PBNA and LTI for 14 HPV types were evaluated in cynomolgus monkeys vaccinated with high doses of the vaccine in Study #2 (370 and 1110 μg total antigen content as shown in Table 1). The results are shown in Figure 4 (and Table S3). The data showed that PBNA and LTI had a good correlation for all HPV types, except HPV 18 and HPV 56 relatively lower, but still clearly significant correlations with all *p* values less than 0.0001.

In Figure 4, there was a high correlation of the immunogenicity between PBNA and LTI for HPV 6, 11, 16, 31, 33, 45, 52, and 58 (at least 0.758). As for HPV 18, the correlation seemed relatively lower (*r* = 0.638), but the immunogenicity data from the two methods were still clearly correlated. This correlation is significant even with the limited size and scope of the sampling (*N* = 36). The data were presented for the new 5 HPV types (HPV 35, 39, 51, 56, and 59) that were not presented in Gardasil 9, and they were all shown with high correlation (at least 0.709), except HPV56 (*r* = 0.637), where the data spreading in neutralization concentration range was limited, but supporting the presence of correlation.

**Figure 5:**
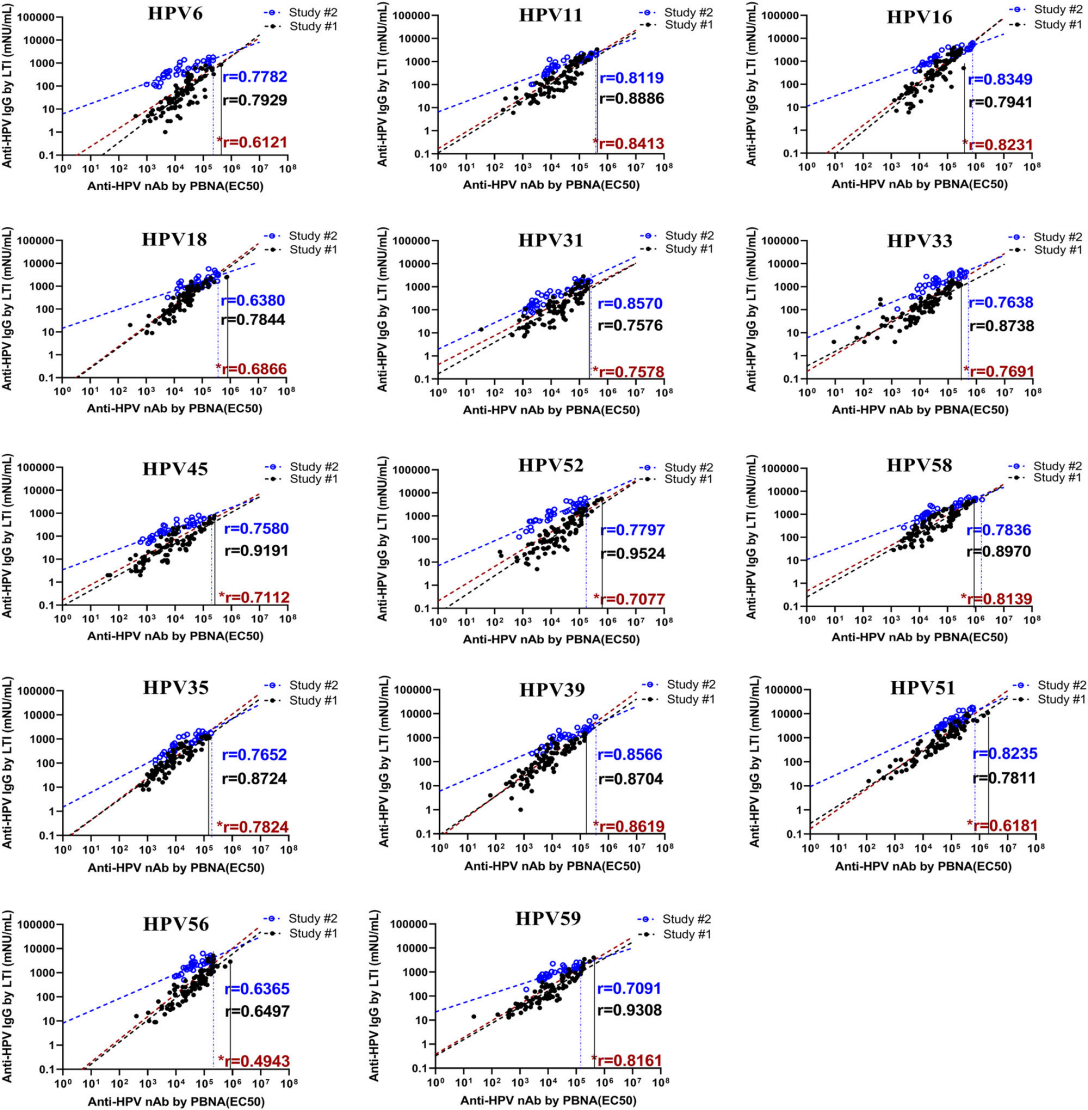
Scatter plots for the data between the PBNA titers (EC_**50**_) and specific total IgG concentration in both studies. ● in black, Study #1; ○ in blue, Study #2; *r* represents the correlation coefficient for which blue represents the data from Study #1 (*N* = 96), black represents the data from Study #2 (*N* = 36), and r in red and marked with * represents the total data from both studies (*N* = 132); the slash dotted lines represents linear regression or concordance, black represents Study #1, blue represents the Study #2, and red represents both studies; vertical solid line in black represents the highest titer of PBNA in Study #1, vertical dotted line in blue represents the highest titer of PBNA in Study #2. IgG, immunoglobulin G; PBNA, pseudovirion‐based neutralization assay

Finally, we combined all the data of the two studies for the correlation analysis, the results of which showed that the immunogenicity data from the PBNA and LTI methods were significantly correlated for all the 14 HPV types (*p* < 0.0001) **(**Figure 5 shown here and Table S4). However, it was observed that the combined correlation coefficient of HPV 6, 45, 51, 52, and 56 from the two studies was relatively lower than those observed in the analysis of each study separately, indicating that there could be some different influencing factors between the studies with different immunization doses which might have caused deviation of the correlation (comparing the blue line and black line). For other HPV types, even with the deviations still present, the influence of the combined analysis was not significant.

## 4 DISCUSSION

For the assessment of immunogenicity of multivalent HPV vaccines from large cohort pivotal or efficacy studies where it is challenging to cover 100% or a large portion of testing subjects with the PBNA within a relatively limited time and resources, LTI assay can be considered as a viable replacement method for the immunogenicity evaluation. In a bivalent HPV vaccine clinical studies, it has been demonstrated that the correlation between the direct‐binding ELISA and PBNA is high for HPV16 and 18, and the high correlation is maintained over time, which supports that the proportion of the neutralization antibody in the total serum IgG is also maintained with time.^11^ For HPV vaccines, the PBNA method is considered “the gold standard” by certain regulatory authorities. However, Cervarix® marketed by GSK, and Gardasil® and Gardasil 9® marketed by Merck took different approaches, using either the total IgG based ELISA or the competitive (with a single neutralizing antibody for each HPV type) Luminex‐based IgG assay (cLIA) to evaluate protective potential of antibodies induced by the vaccines in clinical studies instead of PBNA. The strategies have been well accepted by FDA and EMA.

We reported here a new method for HPV type‐specific total IgG that we think has many advantages in supporting multivalent HPV vaccine development. Compared with an ELISA, the Luminex‐based immunological assays are more sensitive, reproducible, and amenable to high‐throughput testing especially for scarce cross‐reaction during multiple subtypes of vaccines, which will shorten data turn‐around time and save overall cost, and reduce technical, facility, and human resources for the testing. LTI assay is an antibody direct binding assay that measures the total IgG binding to the specific antigens (or VLPs), so that it is a broader measure of the immunogenicity resulting in increased sensitivity, given that the antigens (or VLPs) used in the assays are of high‐quality and representative. However, cLIA demonstrated by Merck is an assay to evaluate the limited neutralizing antibodies which has high specificity at the expense of reduced sensitivity and immunological coverage.^18^ The research on Cervarix® clinical immunogenicity assessment indicated that both direct‐binding ELISA (with the same principle as LTI assay) and single epitope‐based inhibition ELISA (with the same principle as cLIA) showed good correlation with PBNA in clinical studies, with the correlation coefficient from 0.70 to 0.94 for direct‐binding ELISA and from 0.57 to 0.96 for single epitope‐based inhibition ELISA, respectively.^11^ The data showed that both LTI assay and cLIA could be alternative methods to the PBNA for immunogenicity assessment in clinical studies. However, cLIA is limited by single PE conjugated neutralizing mAb and will likely miss many other antibodies in the serum that recognize different epitopes on the VLPs, and the cLIA method could potentially produce “false negative result”. Therefore, LTI could be a more suitable and alternative method to assess the overall vaccine‐induced immune response over time.^18^

There are several reported studies on the correlation analysis between PBNA and the other methods to evaluate protective antibodies in humans as mentioned above, but no such studies have been reported for cynomolgus monkeys covering upto 14 HPV types within one test. Here, we demonstrated the correlation between PBNA and LTI testing of postimmunization sera in the immunogenicity assessment in monkeys and our findings support the use of LTI assay in clinical settings, and, therefore, with the added advantage of the multiplex for the Luminex technology, it is possible to cover as many testing needs as desired, providing a truly suitable supplemental or replacement method for PBNA in both nonclinical and clinical pharmacodynamics studies. We have initiated the method validation work for human sera for the purpose of supporting Phase III efficacy study and immunological bridging studies of various age and ethnic groups in the future. Those works will be reported as well to further confirm the concepts reported here.

In nonhuman primate studies, HPV VLPs have been tested and proved that repeated dosing with as low as 10 μg per dose was able to induce immune responses to reach a plateau.^19^ In this study, we also compared vaccinated animals with different doses and intervals, starting with 37 μg HPV VLPs (i.e., 3 and 6 μg for HPV 6 and 16, 4 μg for both HPV 11 and HPV 18, and 2 μg for all other HPV types) in Study #1 for 3‐time immunization, and 370 μg (i.e., 10 times the individual VLP dosing over the 37 μg dose)/1110 μg (i.e., 30 times the individual VLP dosing over the 37 μg dose) HPV VLPs in Study #2 for 4‐time immunization.

The data in Figure 5 show that the highest titers of PBNA for HPV 6, 11, 18, 45, 52, 51, 56, and 59 in monkeys immunized by the low dose in Study #1 (colored black) are higher than or equal to those of the high‐dose groups in Study #2 (colored blue), almost 10 times of the low dose in Study #1, and the highest titers of PBNA for the other HPV types in Study #1 are very close to those in Study #2. These comparisons indicate that at the low‐dose level, the humoral immunity response in monkeys has approached a plateau. It means that a higher dose cannot provide more protection to the immune system compared with the low dose, shown by the similar peak levels of neutralizing antibodies titers. For all 14 types, there is a relatively higher proportion of the neutralizing antibodies in Study #1 compared with Study #2, especially for HPV 6, 45, 51, 52, and 56 which showed an obvious deviation (reduced titers) with high dose in Study #2. The differences between the two studies (Studies #1 and #2) are not only in the dosage levels but may also be from the difference in immune procedures and products to immunize. These differences would induce differences in the antibody level, but would not influence the correlation assessment to a large extent. The correlation coefficients combining the two studies for 14 HPV types are still relatively good, still adequately supporting the usage of LTI assay in clinical studies. Considering that the products with the improved bioprocess will be used in clinical studies, the limited variation would not be a concern in the future. As shown by this study, the correlation between LTI assay and PBNA was demonstrated, LTI method could be used as an alternative method for clinical immunogenicity assessment.

## 5 CONCLUSION

To support a new VLPs based 14‐valent HPV vaccine development, a new high throughput method (LTI) was developed, and its value and utility were confirmed through the comparative assessment of the immunogenicity in cynomolgus monkeys with the PBNA method. The results and analysis from the monkey studies confirm that LTI with higher throughput, sensitivity, and precision is a new dependable replacement analysis method for the PBNA supporting immunogenicity evaluation in clinical studies, especially for efficacy assessment of multivalent HPV vaccines with large study size and longer duration.

## AUTHOR CONTRIBUTIONS

**Liangzhi Xie and Hongbin Qiu**: conceived the study and provided overall guidance. **Lei Bei and Xiao Zhang**: wrote the original draft. **Lei Bei, Xiao Zhang, Dan Meng, Shuman Gao, and Jilei Jia**: conducted the studies and analysis on data. **Chunxia Luo, Dandan Zhao, and Xuefeng Li**: helped supervise the project. All authors contributed to revising the draft, providing final confirmation of the revised version, and being responsible for all aspects of the work.

## CONFLICTS OF INTEREST

The authors declare no conflicts of interest.

## ETHICS STATEMENT

There is no experimental work with humans included in this article. All animal studies were performed in accordance with the Guide for the Care and Use of Laboratory Animals and approved by the Institutional Animal Care and Use Committee (IACUC).

## Supporting information

Supplementary information.

Supplementary information.

Supplementary information.

Supplementary information.

## Data Availability

The data that support the findings of this study are available from the corresponding author upon reasonable request.
