# Supplementary figures and images for: Immunogenicity correlation in cynomolgus monkeys between Luminex‐based total IgG immunoassay and pseudovirion‐based neutralization assay for a 14‐valent recombinant human papillomavirus vaccine
Source: J Med Virol. 2022 Apr 21;94(8):3946–55. doi: 10.1002/jmv.27763 (PMC9322417; doi:10.1002/jmv.27763)

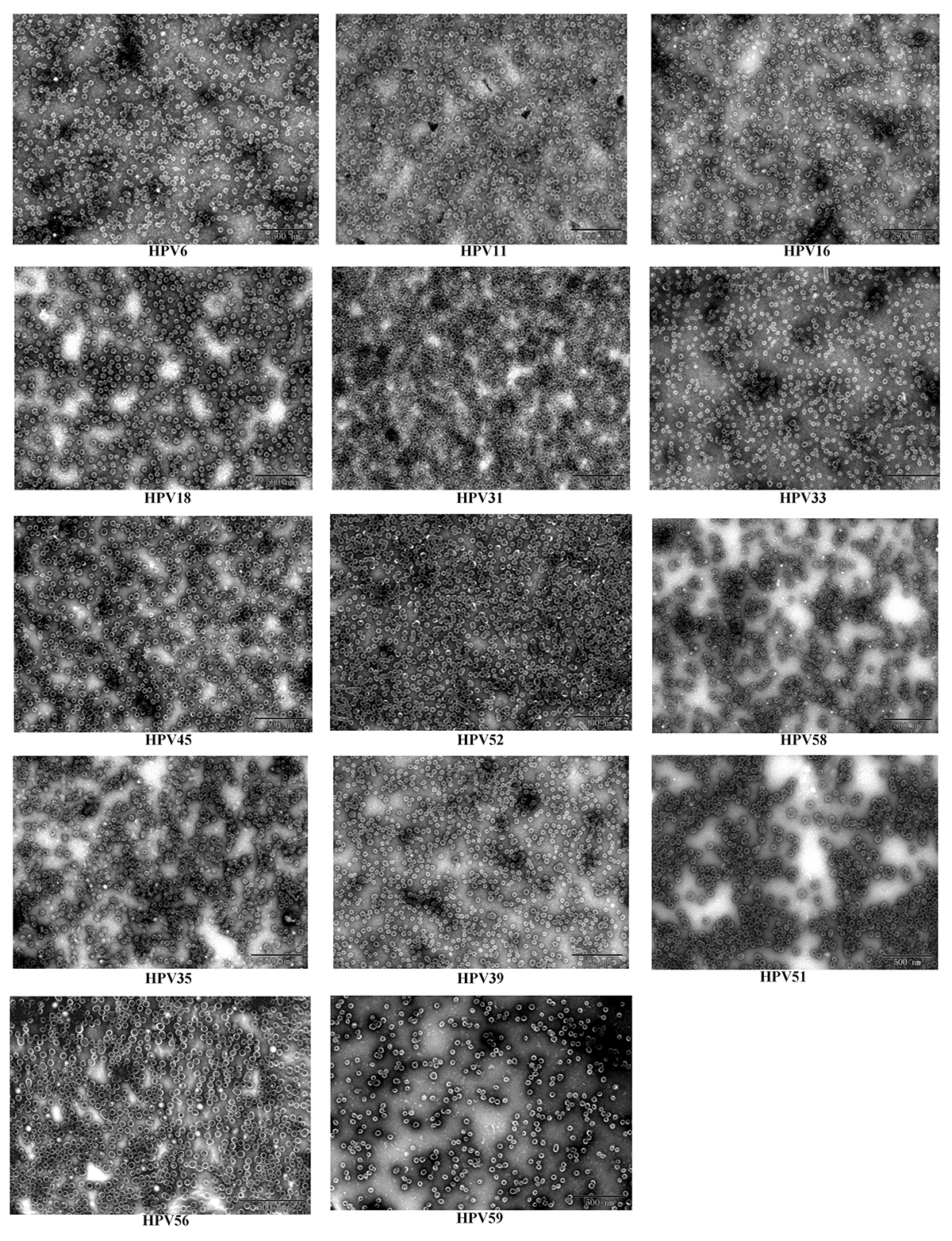

Supplement: Supplementary file 1 — Supplementary information. [file JMV-94--s001.tif]
