# Supplementary material for: Immunogenicity correlation in cynomolgus monkeys between Luminex‐based total IgG immunoassay and pseudovirion‐based neutralization assay for a 14‐valent recombinant human papillomavirus vaccine
Source: J Med Virol. 2022 Apr 21;94(8):3946–55. doi: 10.1002/jmv.27763 (PMC9322417; doi:10.1002/jmv.27763)
Supplement: Supplementary file 2 — Supplementary information. [file JMV-94--s002.pdf]

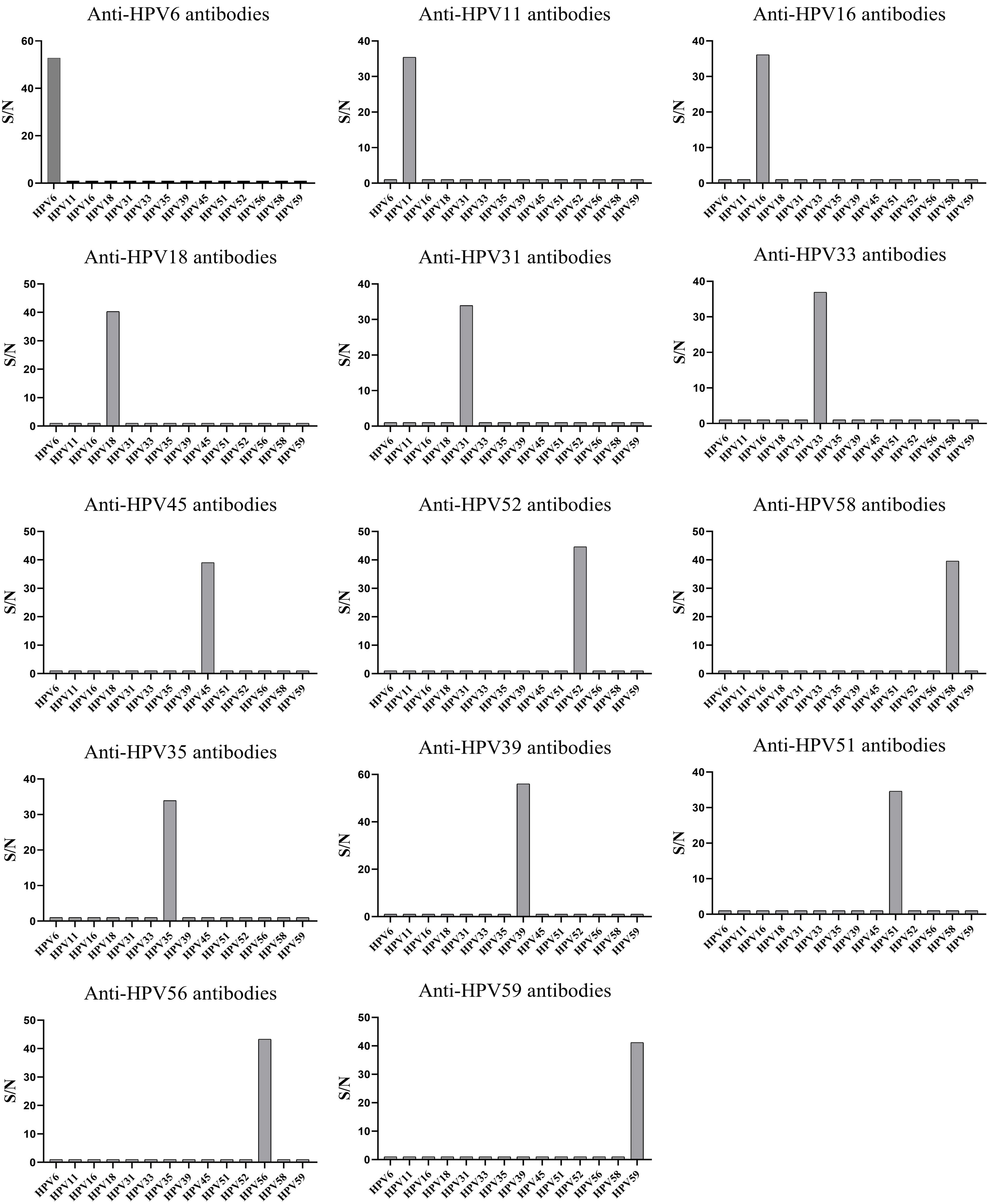
Anti-HPV6 antibodies
Anti-HPV11 antibodies
Anti-HPV16 antibodies
Anti-HPV18 antibodies
Anti-HPV31 antibodies
Anti-HPV33 antibodies
Anti-HPV45 antibodies
Anti-HPV52 antibodies
Anti-HPV58 antibodies
Anti-HPV35 antibodies
Anti-HPV39 antibodies
Anti-HPV51 antibodies
Anti-HPV56 antibodies
Anti-HPV59 antibodies
S/N
HPV6 HPV11 HPV16 HPV18 HPV31 HPV33 HPV35 HPV39 HPV45 HPV51 HPV52 HPV56 HPV58 HPV59
