# Supplementary material for: Immunogenicity correlation in cynomolgus monkeys between Luminex‐based total IgG immunoassay and pseudovirion‐based neutralization assay for a 14‐valent recombinant human papillomavirus vaccine
Source: J Med Virol. 2022 Apr 21;94(8):3946–55. doi: 10.1002/jmv.27763 (PMC9322417; doi:10.1002/jmv.27763)
Supplement: Supplementary file 3 — Supplementary information. [file JMV-94--s003.doc]

Supplemental Information for

**Manuscript ID JMV-22-15094**

**Immunogenicity correlation in cynomolgus monkeys between Luminex-based total IgG immunoassay and** **Pseudovirion-based neutralization assay for a 14-valent recombinant human papillomavirus vaccine**

Lei Bei^1#^, Xiao Zhang^2#^, Dan Meng^2^, Shuman Gao^2^, Jilei Jia^2^, Dandan Zhao^2^, Chunxia Luo^2^, Xuefeng Li^2^, Hongbin Qiu^1^*, Liangzhi Xie^2^*

* Corresponding Authors: Liangzhi Xie, Hongbin Qiu,

^#^ Equal contribution

**This document file includes:**

Figures S1 to S2

Tables S1 to S4

**Methods**

**Electron Microscopy**

Tungsten Filament Transmission Electron Microscope was used in this study to assess the conformational integrity of each VLP type. It is an electron microscope with a tungsten filament as an electron gun. The accelerated and concentrated electron beam is used as a light source and projected onto the sample. The electrons collide with the atoms in the sample and change their direction, thereby producing solid angle scattering. The size of the scattering angle is related to the density and thickness of the sample, so images with different brightness and darkness can be formed, and the image will finally be displayed on the imaging device after being enlarged and focused. The tungsten filament transmission electron microscope (model: JEM 1200EX) produced by JEOL Corporation of Japan was used, and the accelerating voltage was 120kv. The electron microscope was photographed by the following operations: 1) Draw a drop of the sample (about 30 μL) onto the carbon-coated support film copper mesh for 2 min. Absorb excess solution with a pointed filter paper to dry; 2) After staining with uranyl acetate dye solution for 90s, clamp it onto filter paper to dry; 3) Put the copper mesh on the sample handle, observe and shoot a photograph. The particle size and three-dimensional structure of the SCT1000 drug substance were analyzed. The typical results are shown in **Figure S1**.

**Type-specificity evaluation of the VLPs using specific monoclonal neutralizing antibodies**

We have developed in house the 14 type-specific and neutralizing monoclonal antibodies to identify each type of VLPs, suitable for confirming good structural and functional suitability for vaccine development. The specificity of the VLPs was analyzed by enzyme-linked immunosorbent assay (ELISA). 14 types of VLPs are individually combined with the solid phase carrier to form solid phase antibodies, then washed to remove unbound antigens and impurities, and 14 types of specific antibodies against different types of VLPs are separately added to combine with each solid phase antigen. A solid-phase antigen-antibody complex is formed; then horseradish peroxidase-labeled detection antibody (goat anti-rabbit IgG (Fc)/HRP) is added, the antibody on the solid-phase immune complex is bound to the enzyme-labeled antibody, and the substrate is added. The enzyme on the solid phase catalyzes the substrate into a colored product. The absorbance is read at a wavelength of 450 nm, and the signal-to-noise ratio (S/N, Sample/NC) in the sample is analyzed. Among them, S/N is the ratio of the absorbance value of the sample to the absorbance value of the blank control. When S/N ≥ 3, it is indicated as positive. When the anti-VLP antibody recognizes a specific VLP, S/N ≥ 3, indicating that the anti-VLP antibody can specifically recognize a specific VLP. The data are shown in **Figure S2**.

**Type-specificity evaluation of the VLPs in mice**

To evaluate type specificity of the post immunization serum for the VLPs, each type of VLPs of the 14-valent vaccine was used to immunize 3 mice on Day 0, 7 and 21 individually, and the serum was collected on Day 28 to be evaluated for EC_50_ by PBNA. The specificity of the pseudoviruses used had been verified by qPCR with no cross contamination between different types. The results of PBNA showed that there were no obvious cross-reaction between different types of VLPs except for slight cross-reaction between HPV 6 and HPV 11 as shown in **Table S1**.

**Figure S1. The structure of the 14-valent VLPs drug substance analyzed by electron microscope**

**Figure S2. The specificity of each type of VLPs identified by using the verified type-specific antibody.** When the anti-VLP antibody recognizes a specific VLP, S/N ≥ 3, indicating that the anti-VLP antibody can specifically recognize a specific VLP.

**Table S1. Specificity detection of 14-valent vaccine by PBNA after immunization of single type VLP to mice (n=3)**

**Table S2. Pearson correlation coefficient and significance (2 tailed) between PBNA titers (EC_50_) and specific total IgG concentration at all time-points in Study #1.**

**Table S3. Pearson correlation coefficient and significance (2 tailed) between PBNA titers (EC_50_) and specific total IgG concentrations at all time-points in Study #2.**

**Table S4. Pearson correlation coefficient and significance (2 tailed) between PBNA titers (EC_50_) and specific total IgG concentration at all time-points in both studies.**
