# Supplementary material for: Immunogenicity correlation in cynomolgus monkeys between Luminex‐based total IgG immunoassay and pseudovirion‐based neutralization assay for a 14‐valent recombinant human papillomavirus vaccine
Source: J Med Virol. 2022 Apr 21;94(8):3946–55. doi: 10.1002/jmv.27763 (PMC9322417; doi:10.1002/jmv.27763)
Supplement: Supplementary file 4 — Supplementary information. [file JMV-94--s004.docx]

**Table S1. Specificity detection of 14-valent vaccine by PBNA after immunization of single type VLP to mice (n=3)**

| **Single type to immunize** | | **HPV6** | **HPV11** | **HPV16** | **HPV18** | **HPV31** | **HPV33** | **HPV45** | **HPV52** | **HPV58** | **HPV35** | **HPV39** | **HPV51** | **HPV56** | **HPV59** |
| --- | --- | --- | --- | --- | --- | --- | --- | --- | --- | --- | --- | --- | --- | --- | --- |
| **PSV-Type 6^a^** | **1** | **28975** | **4937** | <100^b^ | <100 | <100 | <100 | <100 | <100 | <100 | <100 | <100 | <100 | <100 | <100 |
|  | **2** | **17048** | **15352** | <100 | <100 | <100 | <100 | <100 | <100 | <100 | <100 | <100 | <100 | <100 | <100 |
|  | **3** | **27523** | **52** | <100 | <100 | <100 | <100 | <100 | <100 | <100 | <100 | <100 | <100 | <100 | <100 |
| **PSV-Type 11** | **1** | **814** | **61715** | <100 | <100 | <100 | <100 | <100 | <100 | <100 | <100 | <100 | <100 | <100 | <100 |
|  | **2** | **118** | **55385** | <100 | <100 | <100 | <100 | <100 | <100 | <100 | <100 | <100 | <100 | <100 | <100 |
|  | **3** | **139** | **9749** | <100 | <100 | <100 | <100 | <100 | <100 | <100 | <100 | <100 | <100 | <100 | <100 |
| **PSV-Type 16** | **1** | <100 | <100 | **92738** | <100 | <100 | <100 | <100 | <100 | <100 | <100 | <100 | <100 | <100 | <100 |
|  | **2** | <100 | <100 | **32067** | <100 | <100 | <100 | <100 | <100 | <100 | <100 | <100 | <100 | <100 | <100 |
|  | **3** | <100 | <100 | **18466** | <100 | <100 | <100 | <100 | <100 | <100 | <100 | <100 | <100 | <100 | <100 |
| **PSV-Type 18** | **1** | <100 | <100 | <100 | **33235** | <100 | <100 | <100 | <100 | <100 | <100 | <100 | <100 | <100 | <100 |
|  | **2** | <100 | <100 | <100 | **68155** | <100 | <100 | <100 | <100 | <100 | <100 | <100 | <100 | <100 | <100 |
|  | **3** | <100 | <100 | <100 | **78109** | <100 | <100 | <100 | <100 | <100 | <100 | <100 | <100 | <100 | <100 |
| **PSV-Type 31** | **1** | <100 | <100 | <100 | <100 | **19247** | <100 | <100 | <100 | <100 | <100 | <100 | <100 | <100 | <100 |
|  | **2** | <100 | <100 | <100 | <100 | **64290** | <100 | <100 | <100 | <100 | <100 | <100 | <100 | <100 | <100 |
|  | **3** | <100 | <100 | <100 | <100 | **26263** | <100 | <100 | <100 | <100 | <100 | <100 | <100 | <100 | <100 |
| **PSV-Type 33** | **1** | <20 | <20 | <20 | <20 | <20 | **9630** | <20 | <20 | <20 | <20 | <20 | <20 | <20 | <20 |
|  | **2** | <20 | <20 | <20 | <20 | <20 | **18261** | <20 | <20 | <20 | <20 | <20 | <20 | <20 | <20 |
|  | **3** | <20 | <20 | <20 | <20 | <20 | **4102** | <20 | <20 | <20 | <20 | <20 | <20 | <20 | <20 |
| **PSV-Type 45** | **1** | <20 | <20 | <20 | <20 | <20 | <20 | **726** | <20 | <20 | <20 | <20 | <20 | <20 | <20 |
|  | **2** | <20 | <20 | <20 | <20 | <20 | <20 | **117** | <20 | <20 | <20 | <20 | <20 | <20 | <20 |
|  | **3** | <20 | <20 | <20 | <20 | <20 | <20 | **4191** | <20 | <20 | <20 | <20 | <20 | <20 | <20 |
| **PSV-Type 52** | **1** | <100 | <100 | <100 | <100 | <100 | <100 | <100 | **101092** | <100 | <100 | <100 | <100 | <100 | <100 |
|  | **2** | <100 | <100 | <100 | <100 | <100 | <100 | <100 | **104804** | <100 | <100 | <100 | <100 | <100 | <100 |
|  | **3** | <100 | <100 | <100 | <100 | <100 | <100 | <100 | **51595** | <100 | <100 | <100 | <100 | <100 | <100 |
| **PSV-Type 58** | **1** | <100 | <100 | <100 | <100 | <100 | <100 | <100 | <100 | **42700** | <100 | <100 | <100 | <100 | <100 |
|  | **2** | <100 | <100 | <100 | <100 | <100 | <100 | <100 | <100 | **105661** | <100 | <100 | <100 | <100 | <100 |
|  | **3** | <100 | <100 | <100 | <100 | <100 | <100 | <100 | <100 | **20089** | <100 | <100 | <100 | <100 | <100 |
| **PSV-Type 35** | **1** | <100 | <100 | <100 | <100 | <100 | <100 | <100 | <100 | <100 | **33575** | <100 | <100 | <100 | <100 |
|  | **2** | <100 | <100 | <100 | <100 | <100 | <100 | <100 | <100 | <100 | **24562** | <100 | <100 | <100 | <100 |
|  | **3** | <100 | <100 | <100 | <100 | <100 | <100 | <100 | <100 | <100 | **31060** | <100 | <100 | <100 | <100 |
| **PSV-Type 39** | **1** | <100 | <100 | <100 | <100 | <100 | <100 | <100 | <100 | <100 | <100 | **36232** | <100 | <100 | <100 |
|  | **2** | <100 | <100 | <100 | <100 | <100 | <100 | <100 | <100 | <100 | <100 | **71846** | <100 | <100 | <100 |
|  | **3** | <100 | <100 | <100 | <100 | <100 | <100 | <100 | <100 | <100 | <100 | **26717** | <100 | <100 | <100 |
| **PSV-Type 51** | **1** | <20 | <20 | <20 | <20 | <20 | <20 | <20 | <20 | <20 | <20 | <20 | **15166** | <20 | <20 |
|  | **2** | <20 | <20 | <20 | <20 | <20 | <20 | <20 | <20 | <20 | <20 | <20 | **4324** | <20 | <20 |
|  | **3** | <20 | <20 | <20 | <20 | <20 | <20 | <20 | <20 | <20 | <20 | <20 | **30805** | <20 | <20 |
| **PSV-Type 56** | **1** | <20 | <20 | <20 | <20 | <20 | <20 | <20 | <20 | <20 | <20 | <20 | <20 | **5094** | <20 |
|  | **2** | <20 | <20 | <20 | <20 | <20 | <20 | <20 | <20 | <20 | <20 | <20 | <20 | **13688** | <20 |
|  | **3** | <20 | <20 | <20 | <20 | <20 | <20 | <20 | <20 | <20 | <20 | <20 | <20 | **4234** | <20 |
| **PSV-Type 59** | **1** | <20 | <20 | <20 | <20 | <20 | <20 | <20 | <20 | <20 | <20 | <20 | <20 | <20 | **15489** |
|  | **2** | <20 | <20 | <20 | <20 | <20 | <20 | <20 | <20 | <20 | <20 | <20 | <20 | <20 | **6038** |
|  | **3** | <20 | <20 | <20 | <20 | <20 | <20 | <20 | <20 | <20 | <20 | <20 | <20 | <20 | **8682** |
| **BPV^c^** | **1** | <100 | <100 | <100 | <100 | <100 | <100 | <100 | <100 | <100 | <100 | <100 | <100 | <100 | <100 |
|  | **2** | <100 | <100 | <100 | <100 | <100 | <100 | <100 | <100 | <100 | <100 | <100 | <100 | <100 | <100 |
|  | **3** | <100 | <100 | <100 | <100 | <100 | <100 | <100 | <100 | <100 | <100 | <100 | <100 | <100 | <100 |

a. PSV represents pseudovirus.

b. Lower than lower limit of detection (LLOD).

c. BPV represents Bovine Papillomavirus, which is added as the negative control.

.**Table S2. Pearson correlation coefficient and significance (2 tailed) between PBNA titers (EC_50_) and specific total IgG concentration at all time-points in Study #1.**

| **Pearson correlation** | **HPV type** | **LTI** | | |
| --- | --- | --- | --- | --- |
|  |  | **Correlation coefficient** | **Signal/P value (2 tailed)** | **N** |
| **PBNA** | **6** | 0.793 | < 0.0001 | 96 |
|  | **11** | 0.889 |  |  |
|  | **16** | 0.794 |  |  |
|  | **18** | 0.784 |  |  |
|  | **31** | 0.758 |  |  |
|  | **33** | 0.874 |  |  |
|  | **45** | 0.919 |  |  |
|  | **52** | 0.952 |  |  |
|  | **58** | 0.897 |  |  |
|  | **35** | 0.872 |  |  |
|  | **39** | 0.870 |  |  |
|  | **51** | 0.781 |  |  |
|  | **56** | 0.650 |  |  |
|  | **59** | 0.931 |  |  |

**Table S3. Pearson correlation coefficient and significance (2 tailed) between PBNA titers (EC_50_) and specific total IgG concentrations at all time-points in Study #2.**

| **Pearson correlation** | **HPV Type** | **LTI** | | |
| --- | --- | --- | --- | --- |
|  |  | **Correlation coefficient** | **Signal/P value (2 tailed)** | **N** |
| **PBNA** | **6** | 0.778 | < 0.0001 | 36 |
| **PBNA** | **11** | 0.812 |  |  |
|  | **16** | 0.835 |  |  |
|  | **18** | 0.638 |  |  |
|  | **31** | 0.857 |  |  |
|  | **33** | 0.764 |  |  |
|  | **45** | 0.758 |  |  |
|  | **52** | 0.780 |  |  |
|  | **58** | 0.784 |  |  |
|  | **35** | 0.765 |  |  |
|  | **39** | 0.857 |  |  |
|  | **51** | 0.824 |  |  |
|  | **56** | 0.637 |  |  |
|  | **59** | 0.709 |  |  |

**Table S4. Pearson correlation coefficient and significance (2 tailed) between PBNA titers (EC_50_) and specific total IgG concentration at all time-points in both studies.**

| **Pearson correlation** | **HPV Type** | **LTI** | | |
| --- | --- | --- | --- | --- |
|  |  | **Correlation coefficient** | **Signal/P value (2 tailed)** | **N** |
| **PBNA** | **6** | 0.612 | < 0.0001 | 132 |
|  | **11** | 0.841 |  |  |
|  | **16** | 0.823 |  |  |
|  | **18** | 0.687 |  |  |
|  | **31** | 0.758 |  |  |
|  | **33** | 0.769 |  |  |
|  | **45** | 0.711 |  |  |
|  | **52** | 0.708 |  |  |
|  | **58** | 0.814 |  |  |
|  | **35** | 0.782 |  |  |
|  | **39** | 0.862 |  |  |
|  | **51** | 0.618 |  |  |
|  | **56** | 0.494 |  |  |
|  | **59** | 0.816 |  |  |
